# Successful treatment of a patient with advanced lung adenocarcinoma (EGFR-T790M and C797S cis) with lazertinib: A case report and literature review

**DOI:** 10.3389/fonc.2022.1037964

**Published:** 2023-01-09

**Authors:** Yue Fang, Qiankun Zhang, Weimin Wang, Juanjuan Tong, Xialin Li

**Affiliations:** Hefei Cancer Hospital, Chinese Academy of Sciences, Hefei, China

**Keywords:** lazertinib, lung adenocarcinoma, EGFR-T790M, C797s, case report

## Abstract

Lazertinib has been shown to treat non-small cell lung cancer (NSCLC) patients with EGFR-T790M, Ex19del, and L858R mutations. However, there are still no studies to prove that lazertinib could be used in patients with EGFR-T790M and C797s cis mutations in NSCLC. We report a case of a patient with advanced lung adenocarcinoma with EGFR-T790M and C797s cis mutations who were treated with lazertinib and achieved satisfactory efficacy without serious side effects. And the scratch assay and colony-forming unit assay were performed using lung adenocarcinoma cells from patients, the results showed that both lazertinib and amivantamab could inhibit the proliferation and migration of lung adenocarcinoma cells to some extent, and the inhibitory effect of lazertinib was better than that of amivantamab (*p* < 0. 01), while the inhibitory effect of lazertinib combined with amivantamab was not statistically different from that of lazertinib alone(*p*>0.05). This finding suggests that lazertinib may be an effective treatment option for patients with lung adenocarcinoma presenting with EGFR-T790M and C797s cis mutations.

## Introduction

Lung cancer remains one of the most prevalent malignancies that damage human lives, with approximately 1.7 million deaths from lung cancer worldwide in 2020, as reported by Sung et al (1). Adenocarcinoma of the lung is the most common type of pathology in lung cancer, accounting for approximately 45% of the total number of cases. The five-year survival rate for patients with lung adenocarcinoma is only 15%, as most patients are already in the progressive stage at the time of initial diagnosis (2, 3). Lung adenocarcinoma displays a high degree of heterogeneity, with frequent mutations in multiple genes, including epidermal growth factor receptor (EGFR), anaplastic lymphoma kinase (ALK), v-raf murine sarcoma viral oncogene homolog B1 (BRAF), and Kirsten ratsarcoma viral oncogene homolog (KRAS), which can promote the progression of this cancer. EGFR mutations are the most common gene mutation in patients with lung adenocarcinoma, present in approximately 50-60% of Asian patients; most patients with these mutations have a low survival rate (4).

EGFR-TKIs are a class of targeted drugs that target EGFR mutations, and previous studies have shown that patients are best treated when their sensitive mutation is most frequently found in exon 19 or exon 21 mutations (5). However, first-generation EGFR-TKIs, such as gefitinib, often developed resistance to the drugs after some time (6). It was found that approximately 55% of patients who developed drug resistance had a T790M mutation after first or second-generation EGFR-TKIs treatment, which severely affected survival (7).

Lazertinib, the third-generation EGFR-TKIs approved by the US Food and Drug Administration (FDA) in 2021, was found in previous studies that are effective in improving lung adenocarcinoma patients with T790M mutations (8). However, some patients with lung adenocarcinoma may develop T790M and C797s mutations after taking third-generation EGFR-TKIs, which may lead to drug resistance again. There is still no effective treatment for lung adenocarcinoma patients with both T790M and C797s cis mutations.

Here, we report a case of a patient with advanced lung adenocarcinoma with brain metastases and who had stable disease (SD) after 6 courses of treatment with lazertinib, with no significant side effects.

## Methods

### Primary cells culture

The lung tissue was removed by bronchoscopic puncture under general anesthesia, placed in a sterile dish and transferred to an ultra-clean table, cut into 1-2 mm^3^ pieces, and digested by adding 2 ml of 0.25% trypsin (Beyotime, Shanghai) in a water bath at 37°C for 30 min. Excess digestate was discarded, followed by 3 gentle rinses in PBS (Gibco, USA) and 1 rinse in DMEM (Gibco, USA). Discard excess medium, add 5ml of 10% serum medium, disperse well with a pipette to make a cell suspension, and count under a microscope with a cell counting plate. Cells were placed in DMEM medium with 10% FBS and incubated at 37°C with 5% CO_2_ (9, 10). The cells were recovered and tested by NGS to confirm that the mutation sites were unaltered, and the results showed EGFR T790M and C797s mutations with mutation frequencies of 26.65% and 20.50%, similar to the results of the third NGS test in this patient, and the cells were subsequently used in the experiment.

### Cell scratch assay

Cells were spread evenly in 6-well plates (3x10^4^/well). 3ml of cell culture medium was added and incubated in the incubator. When the cells are 90% fused, discard the medium and rinse gently with PBS twice. Discard the PBS and make vertical lines in the wells with a 200 ml sterile tip. The suspended cells were then gently washed away with PBS and 3 ml of cell culture medium was added. Photographs were taken at 0h and 24h of scratching, respectively, under a microscope (Olympus, China), and the widths were measured and calculated (11).

### Colony-forming unit assay

Digest the cells with 0.25% trypsin for 5 min, add 1 ml of medium to resuspend the cells; evenly spread the mixed cells into a 6-well plate (1×10^3^/well), continue to culture for 10 days, change the medium every 3 days and observe the cell status; After the cloning was completed, the cells were photographed under the microscope, then gently rinsed once with PBS, fixed with 1 mL of 4% paraformaldehyde per well for 30 min, and then gently rinsed with PBS once; shake the six-well plate to cover the staining solution evenly, and incubate for 10 min; then wash the cells three times with PBS, and take pictures for counting after drying (12).

### Statistics

All experiments in this study were repeated three times (n = 3). GraphPad Prism software 7.0 (Beijing, China) was used to generate graphs, and SPSS 23.0 (IBM, USA) was used for the statistical analysis of the data. The *t*-test was used for comparisons between groups. The statistical significance level was set at *p* < 0.05.

### Case report

**Figure 1:**
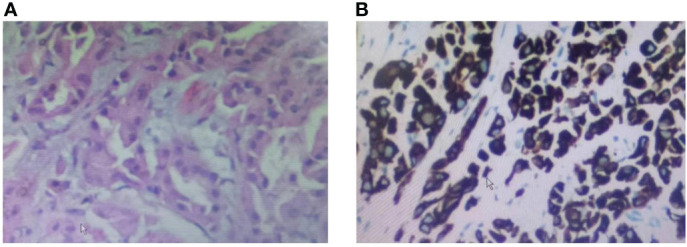
Biopsy pathology of a tumor of the lung lesion. **(A)** HE staining of the patient’s lung lesion, **(B)** IHC staining of the patient’s lung lesion.

The patient was a 37-year-old Chinese female. In December 2017, the patient visited Nanjing Drum Tower Hospital for "pain in the right lower limb". MRI suggested a lower limb femoral lesion. PET-CT results showed that right lung cancer has multiple metastases in the lung, brain, and bone(T3N2M1). Pathology revealed a lung adenocarcinoma lesion, and IHC assay revealed CK7(+), AE1/AE3(+), TTF-1(+), CDX2(-), PAX8(-), NapsinA(-), P63(-), GATA3(-), CK20(-) (**Figures 1A, B**). NSG sequencing suggests EGFR mutations (p.GLY719Cys (22.34%) and EGFR-p.Glu709Val (23.24%) in lung adenocarcinoma.

For further treatment, the patient was transferred to Hefei Cancer Hospital of Chinese academy of science on 11 January, 2018. The physical examination showed the patient's right lung breath sounds were lower than the left, and the right anterior chest was painful on deep inspiration. The patient denied having a family history of oncologic disease. In January 2018, she started treatment with erlotinib (150mg, QD) in combination with bevacizumab (7.5mg/kg). In November 2018, chest CT findings suggested progression of the right lung lesion. The second NGS test was performed in November 2018 and the results suggested EGFR T790M mutation (G719X (24.56%) and T790M (27.81%)) and a change of treatment to osimertinib (80mg, QD).

In November 2019, the patient had a severe headache and a cranial CT showed progression of the brain metastases. Then a change of treatment to bevacizumab + osimertinib and patient headache relief.

**Figure 2:**
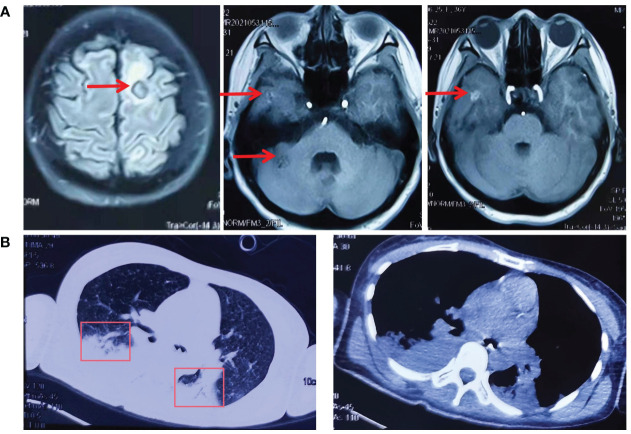
The cranial MR and CT of the chest before and after lazertinib and amivantamab treatment. **(A)** In May 2021, cranial MR showed multiple abnormal signals in the left frontoparietal-occipital lobe, basal ganglia region, thalamus, and right cerebellar hemisphere; **(B)** In July 2021, CT of the chest showed pulmonary atelectasis and pleural effusion.

In May 2021, the patient was readmitted with a severe headache with vomiting. Cranial MR showed multiple abnormal signals in the left frontoparietal-occipital lobe, basal ganglia region, thalamus, and right cerebellar hemisphere (**Figure 2A**). The third NGS test revealed EGFR-T790M and C797s cis mutations(G719C (17.86%), T790M (24.56%) and C797S(21.37%)) and subsequent discontinuation of targeted treatment with osimertinib, which changed to temozolomide + cisplatin + bevacizumab treatment.

On May 28, the patient suffered a sudden onset of unconsciousness with paroxysmal limb twitching and dyspnoea. After tracheal intubation, cranial pressure reduction, sedation, and anti-epileptic treatment, vital signs gradually stabilized. To relieve the patient's symptoms, treatment with gammaglobulin was from April 3rd to 7th. Afterward, the patient's state of consciousness and limb weakness improved compared to before. On June 19, CT of the chest showed pulmonary atelectasis and pleural effusion (**Figure 2B**).

**Figure 3:**
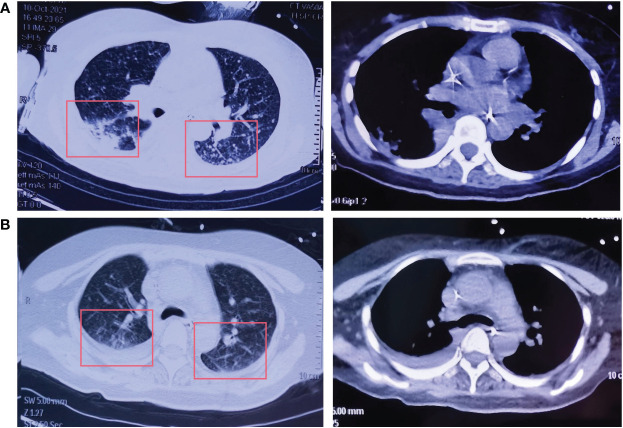
The CT of the chest after lazertinib and amivantamab treatment. **(A)** Increased size and number of diffuse nodules in both lungs on chest CT after amivantamab alone on October 9, 2021; **(B)** Decreased size and number of diffuse nodules in both lungs on chest CT after lazertinib in combination with amivantamab on December 30, 2021.

**Figure 4:**
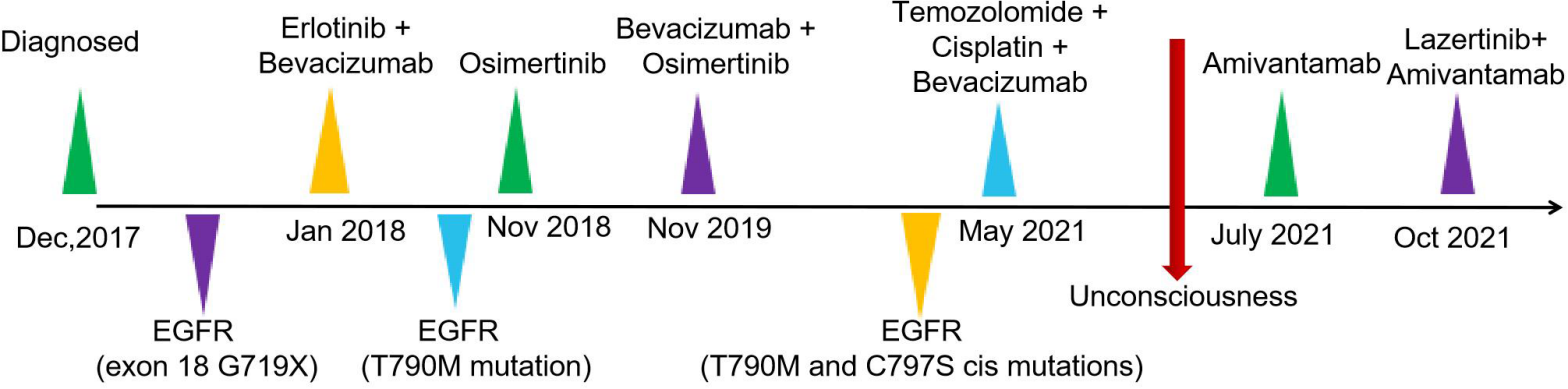
Timeline of treatment.

Amivantamab (350mg/d) single-drug anti-tumor therapy for 6 cycles begins on July 19, 2021. On October 9, CT of the chest showed an increase in the size and number of diffuse nodules in both lungs, suggesting progression of the disease (**Figure 3A**). On October 19, treatment with lazertinib in combination with amivantamab (240mg QD + 700mg d1) was started. The patient's headache subsided, and a chest CT showed a reduction in lung lesions, improved relief of atelectasis, and a reduction in lymph nodes in the lungs on December 30, 2021. The condition was disease-stable (**Figure 3B**). Until our last follow-up in July 2022, the patient was still living and without serious adverse effects. Statistically, the patient had an OS of 54 months **Figure 4**.

## Discussion

Lazertinib (YH25448) is a new, highly potent third-generation EGFR-TKI with good anti-tumor effects in patients with single (Ex19del, L858R, T790M) and dual (Ex19del/T790M and L858R/T790M) EGFR mutations (13, 14).

In a phase 1-2 clinical study by Ahn (15) et al. 127 patients with non-small cell lung cancer with EGFR (L858R, exon 19 deletions, G719X, or L861Q) mutations were enrolled, and approximately 54% of these patients achieved an objective response rate after oral lazertinib.

In addition, in a subgroup analysis of 127 patients divided into 108 patients with T790M-positive mutations and 19 patients with T790M-negative mutations, the objective response rate was approximately 64% in the T790M-positive group and 37% in the negative group after treatment with lazertinib and no significant side-effects. This study confirmed that lazertinib is effective in treating patients with non-small cell lung cancer and is more efficient in patients with T790M mutations. In a study exploring the cardiac safety of lazertinib, a total of 181 patients with EGFR mutation-positive advanced NSCLC treated with lazertinib were enrolled, and the result showed that lazertinib did not cause serious cardiotoxicity (16). The above findings suggest that lazertinib is effective in the treatment of NSCLC patients with the T790M mutation without serious toxic effects. In addition, the previous study has also confirmed the anti-tumor effects of lazertinib *in vitro* studies. To further understand the content of previous studies related to lazertinib, we have summarized general information about patients involved in these studies in **Table 1**, such as race and gender . In addition, in the study by Yun (14) et al. lazertinib was found to be effective in blocking EGFR and downstream signaling pathways in lung cancer cells, and in the nude mouse transplant tumor model, lazertinib was more effective than osimertinib in anti-tumor progression by using the same concentration.

**Table 1:** Previous clinical studies related to lazertinib.

Authors	Year of publication	Number of participants	Race	Median age/age	Sex	Treatment regiem	Ending points
Ahn, et al (15)	2019	127	Asian	NA	Female and male	lazertinib	ORR and AEs
Jang, et al (16)	2021	181	Asian	62	Female and male	lazertinib	Cardiac-related AEs
Park, et al (17)	2020	1	Asian	38	Male	lazertinib	NA

NA, unknown.

We report a case of a female patient with advanced lung adenocarcinoma who developed T790M and C797s cis mutations after gefitinib, chemotherapy, and osimertinib, and whose disease continued to progress after three months of amivantamab, while tumor progression was significantly inhibited after the combination of lazertinib. In contrast to our report, Park et al (17). reported a patient with EGFR-T790M and C797s cis mutations after treatment of lazertinib. This is contrary to the results of our report, with such a little information that the authors provided, we are unable to determine exactly what caused this patient to be resistant to lazertinib. But in cell viability assay and western blot assay, the cells drived from patient were also shown a c-MET mutation and resistence to savolitinib(c-Met inhibitor). As reported in previous studies, c-MET mutation is one of the common bypass mutations in lung adenocarcinoma patients with EGFR mutation who develop drug resistance (18). Therefore, we speculate that the patient had a T790M and C757s mutation along with a c-MET mutation, which led to stimulated resistance to lazertinib. However, this conjecture still needs to be further confirmed by extensive *in vivo* and vitro experiments.

**Figure 5:**
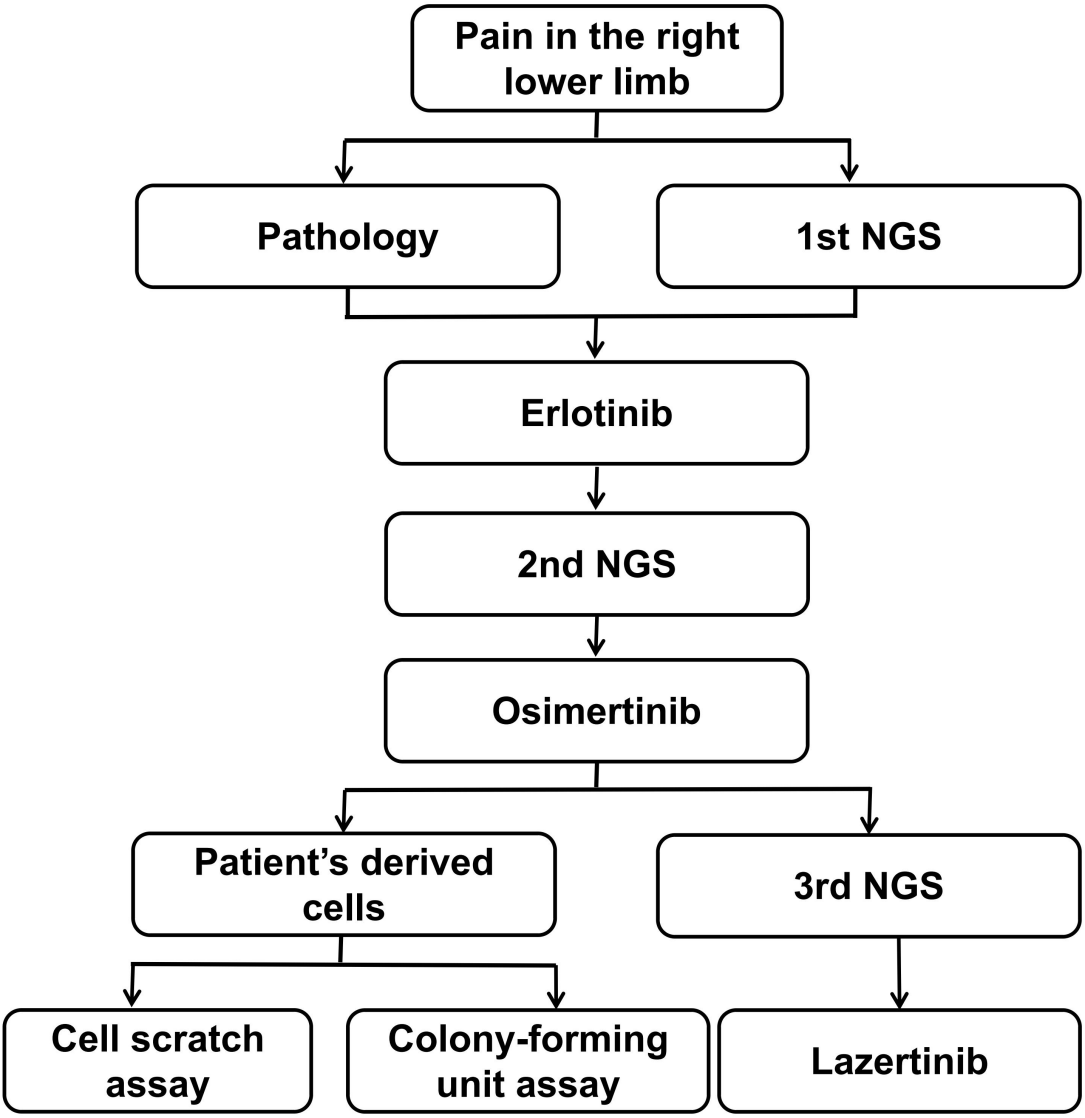
Figure of analysis process.

In our report, the patient was treated with lazertinib in combination with amivantamab. To clarify which of these two drugs exerted an anti-cancer effect, we investigated the inhibitory effects of these two drugs on cell invasion and proliferation by culturing patient-derived lung adenocarcinoma cells (T790M and C797s cis) and cell scratch assay and Colony-forming unit assay. Our analysis process is showing in **Figure 5**.

Con: only patient-derived lung adenocarcinoma cells (T790M and C797s cis); Laz: patient-derived lung adenocarcinoma cells (T790M and C797s cis)+ lazertinib (1 µ/mol); Ami: patient-derived lung adenocarcinoma cells (T790M and C797s cis)+ amivantamab (1 µ/mol); Laz+Ami:patient-derived lung adenocarcinoma cells (T790M and C797s cis)+ lazertinib (1 µ/mol)+amivantamab. ** and ***p< 0.01, 0.001, respectively, compared to the control group.

**Figure 6:**
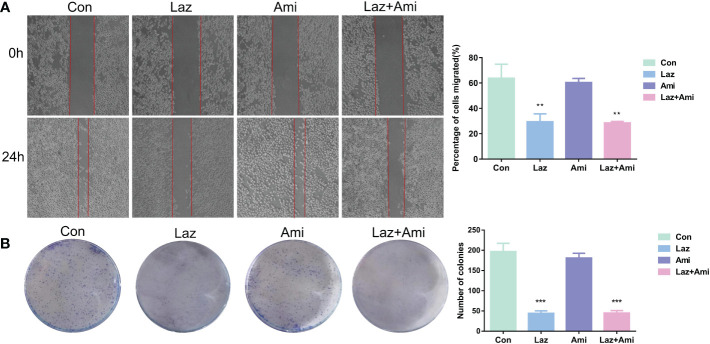
The effect of lazertinib and amivantamab on migration and proliferation of patient’s lung adenocarcinoma cells. **(A)** Scratch assay shows lazertinib inhibits migration ability of lung adenocarcinoma cells better than amivantamab; **(B)** Colony-forming unit assays shows lazertinib inhibits lung adenocarcinoma cell proliferation ability more than amivantamab. *p < 01, ***p < 0.001.

The results showed that both lazertinib and amivantamab could inhibit cell proliferation and migration, and the inhibitory effect of lazertinib was better than that of amivantamab (*p* < 0.01), while the inhibition effect of lazertinib combined with amivantamab was not statistically different from that of lazertinib alone (**Figures 6A, B**, *p* > 0.05). This result suggests that lazertinib is effective in treating lung adenocarcinoma patients with EGFR-T790M and C797s mutations, while the combination of amivantamab did not significantly improve the treatment effect.

Our study is the first to report successful treatment with lazertinib in patients with advanced lung adenocarcinoma with EGFR-T790M and C797s cis mutations and validated this conclusion with *in vitro* experiments. However, our study has some shortcomings, such as: 1. only one patient report; 2. lack of evidence from animal studies. These shortcomings will be improved in our future studies.

## Conclusion

We here report a case of advanced lung adenocarcinoma (T790M and C797s cis mutations) successfully treated with lazertinib with an OS of 54 months, and confirm the therapeutic effect of lazertinib by *in vitro* experiments. This finding is expected to help patients with lung adenocarcinoma who also have T790M and C797s cis mutations choose their treatment subject.

### Patients perspective

I was initially suffering from pain in the right lower limb and was diagnosed with lung cancer in another hospital. Therefore, I came here with the aim of better treatment. Doctor Li and Fang have devised a comprehensive treatment plan for me. One day in 2021, I fell into a coma and Dr Li informed my husband that I might have become drug-resistant again, which led to the non-stop progression of lesions in my lungs and brain. After another genetic test, Dr Fang told me that I had a rare mutation for which there was no targeted drug and suggested I try lazertinib or amivantamab. The doctors then treated me with amivantamab, but unfortunately my disease continued to progress. Dr Li and Dr Fang then treated me with lazertinib and my disease finally stabilized after a few treatments. I am very grateful to the doctors for helping to ease my pain and extend my life. I agreed to share my medical history and Specimens, I have already signed an informed consent form.

## Data availability statement

The raw data supporting the conclusions of this article will be made available by the authors, without undue reservation.

## Ethics statement

The studies involving human participants were reviewed and approved by Medical Ethics Committee of Hefei Cancer Hospital, Chinese Academy of Sciences. The patients/participants provided their written informed consent to participate in this study. Written informed consent was obtained from the individual(s) for the publication of any potentially identifiable images or data included in this article.

## Author contributions

Xl and YF participated in the conception and design of the research; YF and QZ completed the experiment and wrote the article; WW and JT helped to collect information. The final manuscript was reviewed by all authors. All authors contributed to the article and approved the submitted version.
